# A Case of Anti-TIF1γ Antibody-Positive Dermatomyositis Associated With Malignancy

**DOI:** 10.7759/cureus.85930

**Published:** 2025-06-13

**Authors:** Muhammad Yasir, Abdelnassir Abdelgabar, Mohammed Elsayed, Md Nayeem Hasan, Sherif Osman

**Affiliations:** 1 Acute Medicine, Diana, Princess of Wales Hospital, Grimsby, GBR

**Keywords:** anti-transcriptional intermediary factor 1 (tif1), autoantibodies, dermatomyositis, malignancy, paraneoplastic

## Abstract

Dermatomyositis (DM) is a rare acquired autoimmune myopathy characterized by proximal muscle weakness, inflammation, and a typical skin rash. It is considered one of the idiopathic inflammatory myopathies (IIM), a group of heterogeneous systemic diseases that include DM, polymyositis, and inclusion body myositis. A significant portion of patients with IIM, particularly adults, can have an association with malignancy, usually preceded by muscle and skin symptoms.

We report a case of a 67-year-old man who presented to the accident and emergency department with a six-week history of proximal myopathy in the upper and lower limbs and a skin rash. After a series of investigations, the patient was diagnosed with anti-TIF1γ-positive DM and concurrent lung cancer.

DM is a paraneoplastic disorder that should always prompt a search for malignancy. Early detection of anti-TIF1γ autoantibodies can facilitate an early diagnosis of CAD, allowing for prompt therapeutic interventions. Several autoantibodies with different clinical and prognostic significance have been detected in IIM. However, the recently discovered anti-TIF1γ antibodies appear to be strongly associated with malignancy.

## Introduction

Dermatomyositis (DM) is a multisystem connective tissue disorder, which, along with polymyositis (PM) and inclusion body myositis (IBM), forms a heterogeneous group of autoimmune diseases collectively termed idiopathic inflammatory myopathies (IIM) [1]. Based on clinical phenotypes, histological characteristics, organ involvement, and disease course, IIMs are further classified into subtypes [1]. DM can occur in juvenile and adult forms, with more women affected than men. The typical presentation of DM includes proximal muscle weakness and skin manifestations. The classical skin manifestations include a heliotrope rash on the upper eyelids and Gottron’s rash, which are violaceous papules on the metacarpophalangeal and interphalangeal joints. Skin involvement may also affect the sun-exposed parts of the anterior neck and upper chest, termed the V-sign.

The association between IIM and malignancy is well known and has been recognized for a long time [2]. This association is significantly higher with DM than with PM or other forms of myositis, with an estimated fivefold increased risk of cancer compared to the general population [2]. In most cases, cancer develops simultaneously with myositis or within the first year of diagnosis. The risk of cancer development remains for years after the onset of myositis, although it gradually decreases [3].

Circulating autoantibodies are a characteristic laboratory finding in IIM and can be found in up to 80% of patients [4]. They are categorized into two subgroups: myositis-specific autoantibodies (MSA) and myositis-associated antibodies (MAA). MSA includes antibodies against aminoacyl-tRNA synthetases, such as anti-JO-1, anti-PL-7, and anti-PL-12, as well as anti-Mi-2, anti-CADM-140, anti-SAE, and anti-TIF1γ. These MSAs are detected in about 50% of IIM patients. MAA, such as anti-Ku, anti-Ro, anti-La, anti-U1-RNP, and anti-U3RNP, are found in about 20% of myositis patients [5] and other overlapping systemic immune syndromes [6]. These autoantibodies play a role in determining the clinical course, prognosis, response to treatment, and risk of malignancy. An example is the association between interstitial lung disease (ILD) and anti-aminoacyl-tRNA antibodies, as well as between TIF1γ antibodies and malignancy [7].

Genetic factors are thought to contribute to the development of cancer-associated myositis (CAM), particularly in Caucasian populations, where HLA-DQA1*0301 is associated with the presence of anti-p155/140 and TIF1γ autoantibodies, which are considered risks for CAM [8]. It is believed that the abnormal expression of autoantigens in neoplasms can induce cross-reactivity against self-proteins, resulting in paraneoplastic myositis [9]. These autoantigens are overexpressed in certain cancers, such as lung and breast malignancies, but not in healthy tissues.

## Case presentation

A 67-year-old male presented with a six-week history of worsening fatigue, proximal weakness in the upper and lower limbs, and a rash. Initially, he had trouble lifting his arms above shoulder level, followed three weeks later by difficulty climbing stairs and standing from a sitting position. Two weeks prior to the presentation, he developed an erythematous rash with telangiectasia on his face, neck, chest, and hands.

He reported a weight loss of 6 kg over the last two months but denied fever, night sweats, arthralgia, dysphagia, chest pain, cough, or shortness of breath. His medical history included chronic obstructive pulmonary disease, and he was an ex-smoker. There was no family history of autoimmune diseases.

**Figure 1:**
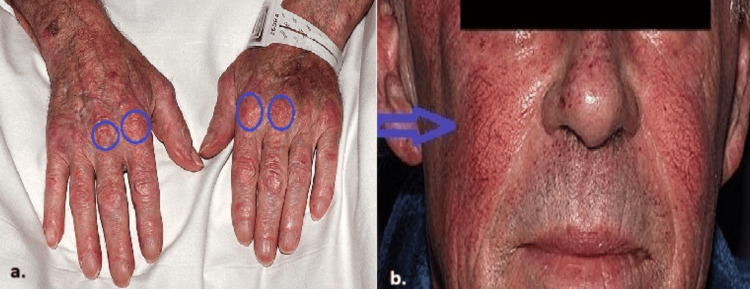
(a) Gottron's papules on both hands (Gottron's sign). (b) Butterfly rash with telangiectasia on both cheeks sparing the nasolabial fold

On examination, all vital signs were normal. An erythematous rash and telangiectasia were noted on the face, neck, and chest, and Gottron's papules were noted on the hands (Figure 1).

Proximal muscle weakness (grade 3/4) was present in the thighs and upper limbs, with intact tendon reflexes and no muscle tenderness. The cardiovascular examination revealed normal heart sounds, and the pulmonary examination noted scattered wheezes without clubbing or fine inspiratory crackles.

Laboratory investigations showed normal blood counts and renal, liver, and bone profiles. C-reactive protein (CRP) was 0.8 mg/L, and erythrocyte sedimentation rate (ESR) was 1 mm/h. Lactate dehydrogenase (LDH) was 190 U/L, and creatine kinase (CK) was 139 U/L. A wide panel of antibodies, including antinuclear antibody (ANA), anti-mitochondrial antibody (AMA), and anti-neutrophil cytoplasmic antibody (ANCA), was negative. The patient was also negative for several myositis-related antibodies, including anti-JO-1 and anti-Mi-2 (Table 1).

**Table 1:** Laboratory data

Test	Result	Reference range
CRP (C-reactive protein)	0.8 mg/L	<5 mg/L
ESR (erythrocyte sedimentation rate)	1 mm/h	Men: 0–15 mm/h, women: 0–20 mm/h
LDH (lactate dehydrogenase)	190 U/L	140-280 U/L
CK (creatine kinase)	139 U/L	26-192 U/L
ANA (antinuclear antibody)	Negative	Negative
AMA (anti-mitochondrial antibody)	Negative	Negative
ANCA (anti-neutrophil cytoplasmic antibody)	Negative	Negative
Anti-JO-1 antibody	Negative	Negative
Anti-Mi-2 antibody	Negative	Negative

**Figure 2:**
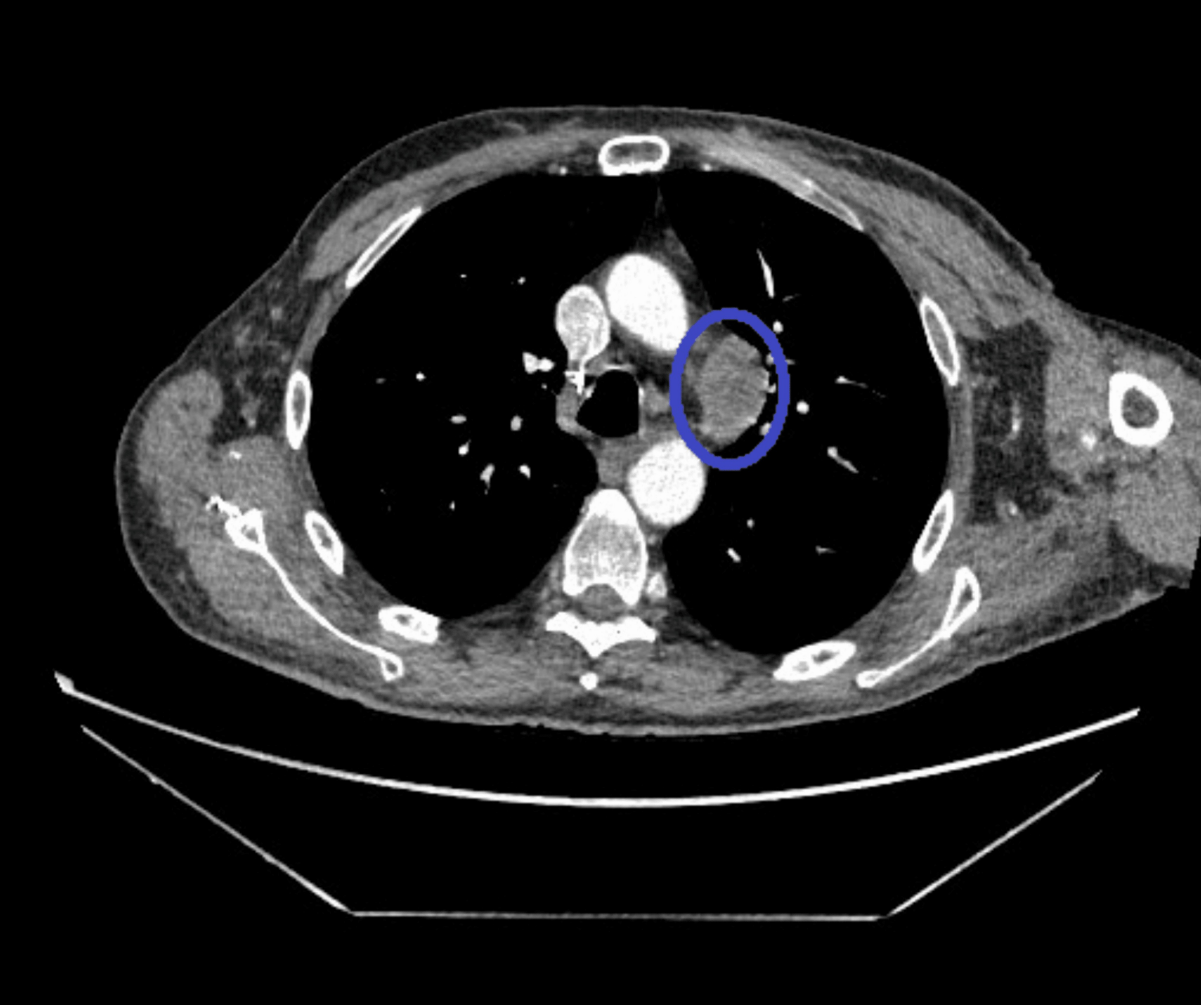
CT chest revealing a prominent perivascular lymph node in the aortopulmonary window CT: computed tomography

A CT scan of the chest, abdomen, and pelvis (Figure 2) revealed a prominent perivascular lymph node measuring 3.6 cm in length and 2.4 cm in short axis in the aortopulmonary window.

**Figure 3:**
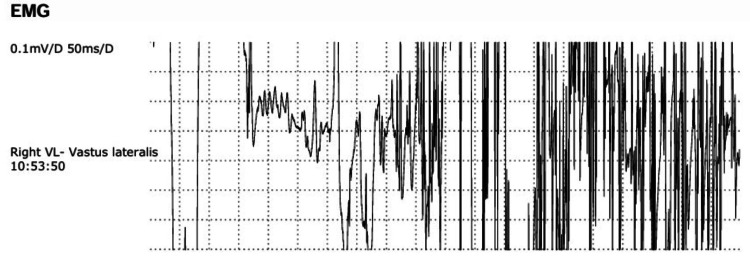
EMG showing features of inflammatory myopathy consistent with PM EMG: electromyography, PM: polymyositis

Electromyography (EMG) showed features of inflammatory myopathy consistent with PM (Figure 3, Table 2).

**Table 2:** EMG findings consistent with inflammatory myopathy (PM) EMG: electromyography, VL: vastus lateralis, VM: vastus medialis, TA: tibialis anterior, Psp: paraspinal, Mid: middle, Fib: fibrillation potentials, PSW: positive sharp waves, Amp: amplitude, Dur: duration, Poly: polyphasic potentials, Stabil: stability, IP: interference pattern, ?myo: possible myopathy, NIL: nothing abnormal detected/no additional notes, PM: polymyositis

Muscle	Interpretation	Fib	PSW	Amp	Dur	Poly	Stabil	IP	Notes
Right deltoid middle	Myopathy	Occasional	Occasional	Short	Short	0	Normal	Normal	NIL
Right biceps	Myopathy	Occasional	Occasional	Short	Short	0	Normal	Normal	NIL
Left cervical Psp Mid	Myopathy	3	3	Short	Short	0	Normal	Normal	NIL
Left upper trapezius	Myopathy	Occasional	Occasional	Short	Short	Few	Normal	Normal	NIL
Left deltoid middle	Myopathy	Occasional	Occasional	Short	Short	Excessive	Normal	Normal	NIL
Right VL	Myopathy	Occasional	3	Short	Short	Excessive	Normal	Normal	NIL
Left VL	Myopathy	3	3	Short	Short	Excessive	Normal	Normal	NIL
Left VM	Normal ?myo	Occasional	Occasional	Normal	Normal	Few	Normal	Normal	NIL
Right VM	Normal ?myo	Occasional	Occasional	Normal	Normal	Few	Normal	Normal	NIL
Right TA	?myo	Occasional	Occasional	Normal	Short	Excessive	Normal	Normal	NIL

**Figure 4:**
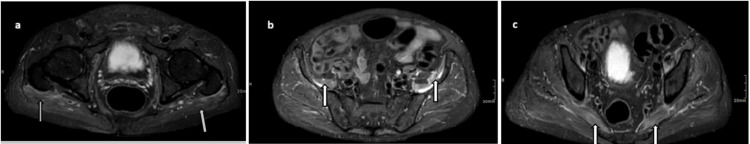
MRI of the femurs showing diffuse, symmetrical hyperintense signal within the gluteus maximus (a), iliopsoas (b), and piriformis (c) muscles bilaterally MRI: magnetic resonance imaging

MRI of the femurs showed diffuse, symmetrical hyperintense signals within the gluteus maximus, iliopsoas, and piriformis muscles bilaterally (Figure 4).

**Figure 5:**
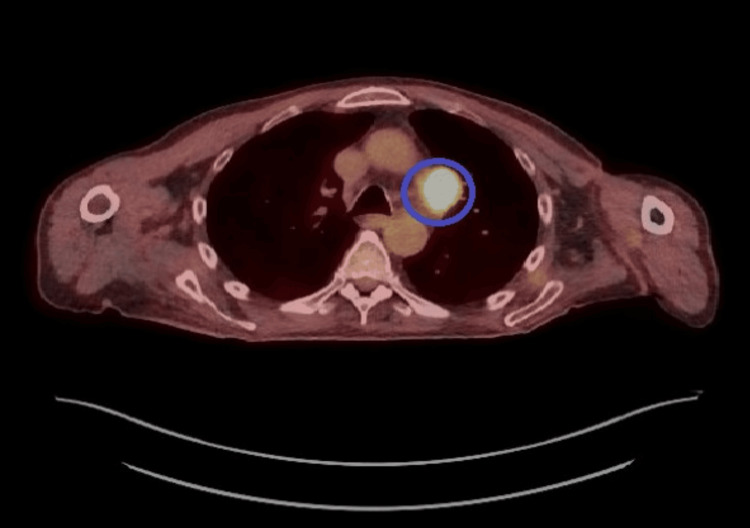
PET scan showing FDG-avid lymph node PET: positron emission tomography, FDG: fluorodeoxyglucose

A PET scan demonstrated fluorodeoxyglucose (FDG)-avid lymph nodes (Figure 5), and endobronchial ultrasound-guided biopsy confirmed small-cell anaplastic carcinoma staging TXN2M1 b; the sample was positive for TIF1γ antibodies.

**Figure 6:**
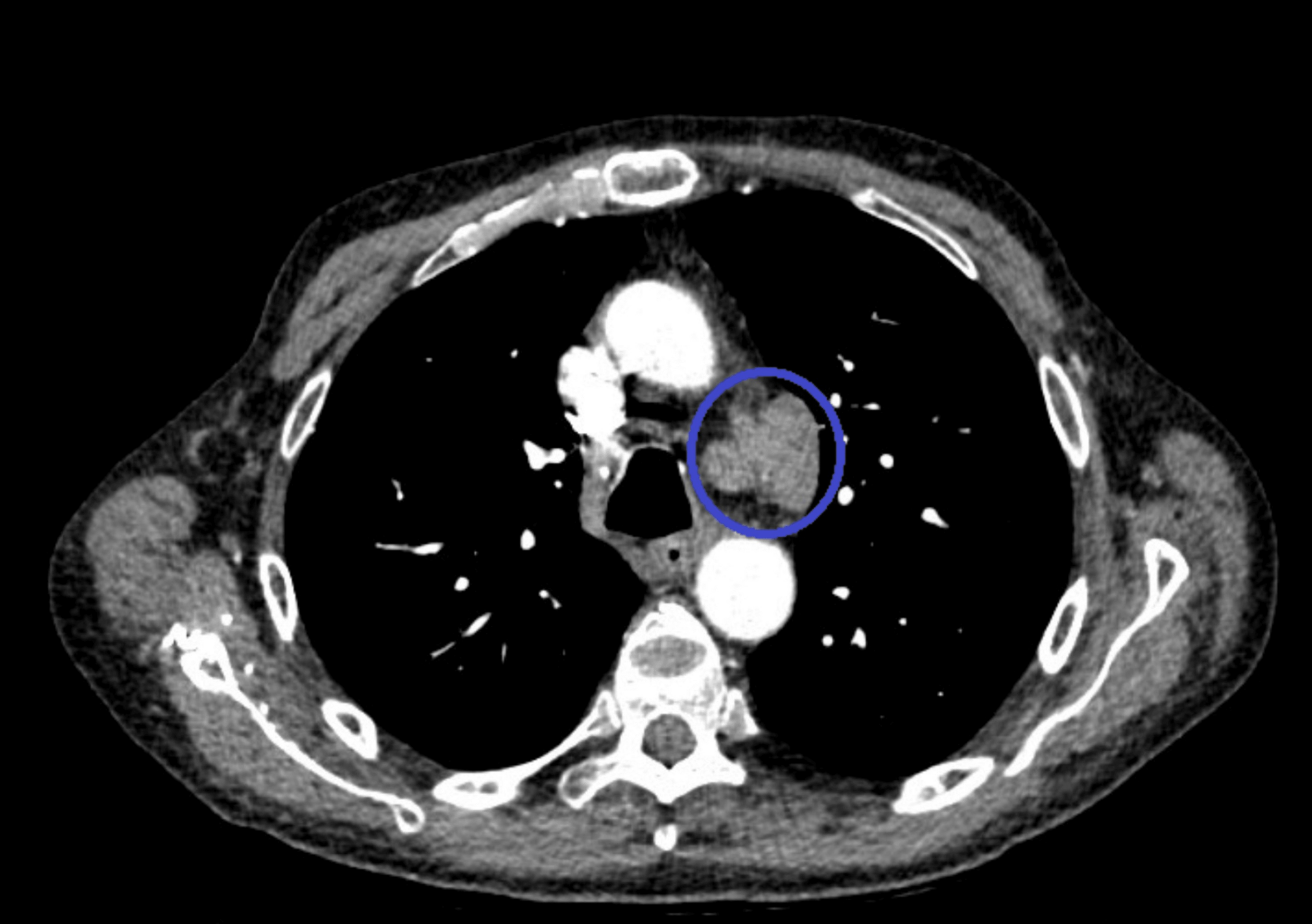
Repeated CT chest one year later showing disease progression CT: computed tomography

The patient was started on 40 mg of prednisolone, along with a proton pump inhibitor, vitamin D, and alendronic acid. He showed rapid improvement and regained independence within two weeks. For his lung cancer, he was referred to oncology and underwent six cycles of chemotherapy (carboplatin/etoposide), which he tolerated well. His steroids were adjusted regularly during treatment. One year later, he was symptom-free on 5 mg of prednisolone, although recent CT scans revealed disease progression (Figure 6). The patient was advised that no further chemotherapy was necessary and sadly passed away a few months later.

## Discussion

The diagnosis of DM is traditionally based on specific criteria, which include symmetrical proximal muscle weakness, muscle biopsy evidence of myositis, increased serum CK levels, characteristic EMG patterns, and typical skin manifestations [10]. Our patient met most of these criteria, resulting in a high likelihood of developing DM. Once a diagnosis of DM is made, every effort should be made to look for cancer in these patients, particularly the higher-risk groups.

The diagnosis of CAM can be considered if it occurs within three years of the initial IIM diagnosis [7]. However, most malignancies occur within the first year following IIM diagnosis, with a gradual decrease in risk over time [11]. CAM is considered to be paraneoplastic based on the temporal coincidence and the relief of the muscle symptoms after the tumor removal. It must be emphasized that myositis and cancer might follow two different courses. The highest risk of malignancy is found in DM, affecting 32% of patients, and to a lesser extent in PM, affecting 15% of patients [12]. The risk of malignancy is noted to be higher in men compared to women and in the elderly compared to the young [2]. Patients with amyopathic DM, patients with typical skin lesions without muscle symptoms, are also frequently observed to have malignancy [13]. Less observed malignancy is noted in patients with antisynthetase syndrome, particularly those with ILD and IIM [7]. The strongest correlation with malignant diseases was reported with anti-TIF1γ-positive patients, with a 9.37-fold higher risk of cancer [2]. An increased risk, albeit to a lesser extent, is also noted with NXP2 and SAE antibodies [2]. This group of patients must be closely monitored for the development of DM-associated malignancy.

Key risk factors for malignancy in DM include diagnosis within the first 12 months, age over 44 years, rapid symptom onset, male sex, presence of dysphagia, cutaneous necrosis, elevated inflammatory markers such as ESR or CRP, and increased CK levels [2]. In contrast, conditions such as ILD, arthritis or arthralgia, and Raynaud’s phenomenon are associated with a lower risk of malignancy [14].

The types of malignancies most commonly associated with DM vary, with nasopharyngeal cancer being the most frequent (22.5-62.5%), followed by lung cancer (8-34%), breast cancer (2.8-24%), colorectal cancer (5.2-14%), lymphatic and hematopoietic cancers (3.6-17%), ovarian cancer (2-11%), prostate cancer (1-9.4%), and gastric cancer (1-6%) [2,15,16].

All DM patients should also be investigated for MAA, of which some are associated with a higher risk of malignancy, such as TIF1γ and NXP-2 antibodies. These autoantibodies are frequently found in patients with CAD, present in 83% of cases, with 52% anti-TIF1γ and 31% anti-NXP-2, making them a valuable tool for screening for malignancy in DM with a sensitivity and specificity of 52% and 92%, respectively [17]. Our patient was positive for the TIF1γ antibody. TIF1γ expression has been suggested to play a role in the TGF-β signaling pathway, which controls cell proliferation, apoptosis, and tumorigenesis [18].

TIF1γ antibody expression has been associated with young age, aggressive tumor grades, more estrogen receptor negativity, and tumors larger than 2 cm, collectively signaling poor prognosis. It has also been observed that TIF1γ antibody is non-overlapping and that the vast majority of TIF1γ antibody-positive patients are negative for the other MSA, as in our patient’s case [19].

A subset of patients with DM, predominantly presenting with the typical cutaneous findings and very minimal muscle symptoms with normal muscle enzymes, is classified as amyopathic DM. Our patient was unique in that, although he experienced significant unexplained muscle symptoms, his muscle enzymes, including CK, were within normal limits. Factors leading to low CK, such as cachexia, steroids, or chemotherapy treatment, chronic liver disease, and immobility, were all absent. Another atypical feature in our patient is that, although low CK levels imply a favorable prognosis in the White population, this was not the case in our patient.

DM is a potentially treatable disease, and systemic corticosteroids remain the mainstay in therapy. In patients who develop side effects or are intolerant to corticosteroids, other immunosuppressive agents can be tried. Targeted cancer treatment, including surgical removal, radiotherapy, or chemotherapy, can also alleviate the symptoms of DM.

## Conclusions

DM is a paraneoplastic disorder that should prompt a thorough search for malignancy, especially in high-risk groups. The presence of TIF1γ antibodies might serve as a potential prognostic marker for increased cancer risk and poor prognosis. Management includes corticosteroids or immunosuppressive agents, in addition to targeted cancer treatments.
